# Supplementary figures and images for: The lunar cycle drives migration of a nocturnal bird
Source: PLoS Biol. 2019 Oct 15;17(10):e3000456. doi: 10.1371/journal.pbio.3000456 (PMC6794068; doi:10.1371/journal.pbio.3000456)

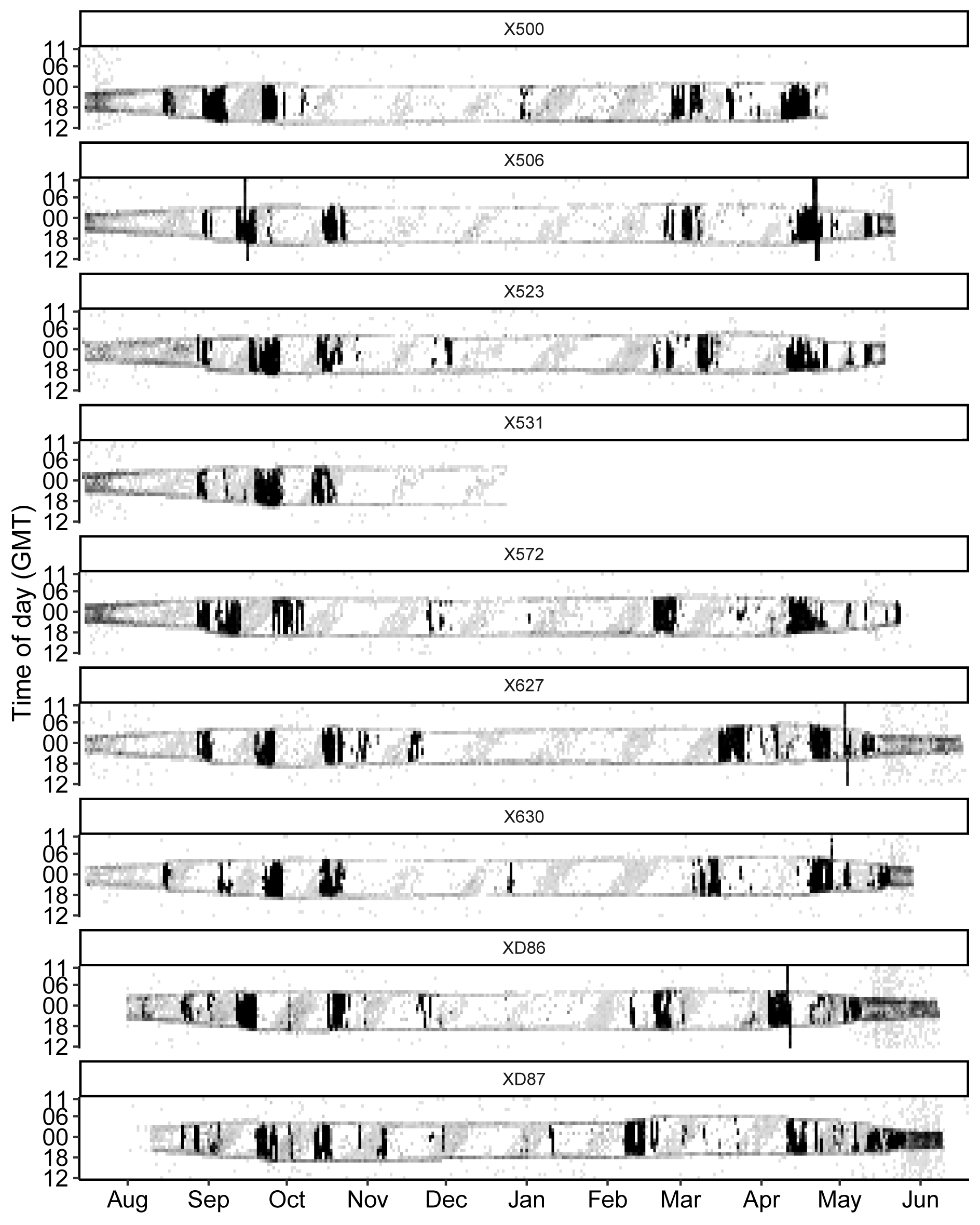

Supplement: S1 Fig — Actogram showing the hourly activity index of all individuals with no (white) to continuous (black) flight activity throughout the nonbreeding season. Gray corresponds to intermittent flight activity representing foraging flights. The birds’ activity is confined to the night and only exceptionally is activity recorded during daylight hours, which demonstrates the species’ crepuscular and nocturnal lifestyle. Black vertical lines represent periods of high activity spanning several hours (indicative of migratory flights), and aggregations of black lines represent consecutive nights of migratory flights. Intermittent flight activity, presumably related to foraging, is concentrated towards dusk and dawn. Following the daily cycle of the moon, intermittent flight activity is also registered during nighttime, resulting in diagonal bands throughout the nonbreeding season. Notice the shorter activity periods from mid-April to May and in August, which reflect the shorter nights at northern latitudes. Individual activity data were recorded from 15 July (or from the date of mounting; individuals XD86 and XD87) to the date of recovery of the tag (or when the tag stopped; individuals X500, X523, X526, and X531). Underlying data are found in S2 Data. MDL, multisensor data logger. (TIF) [file pbio.3000456.s006.tif]

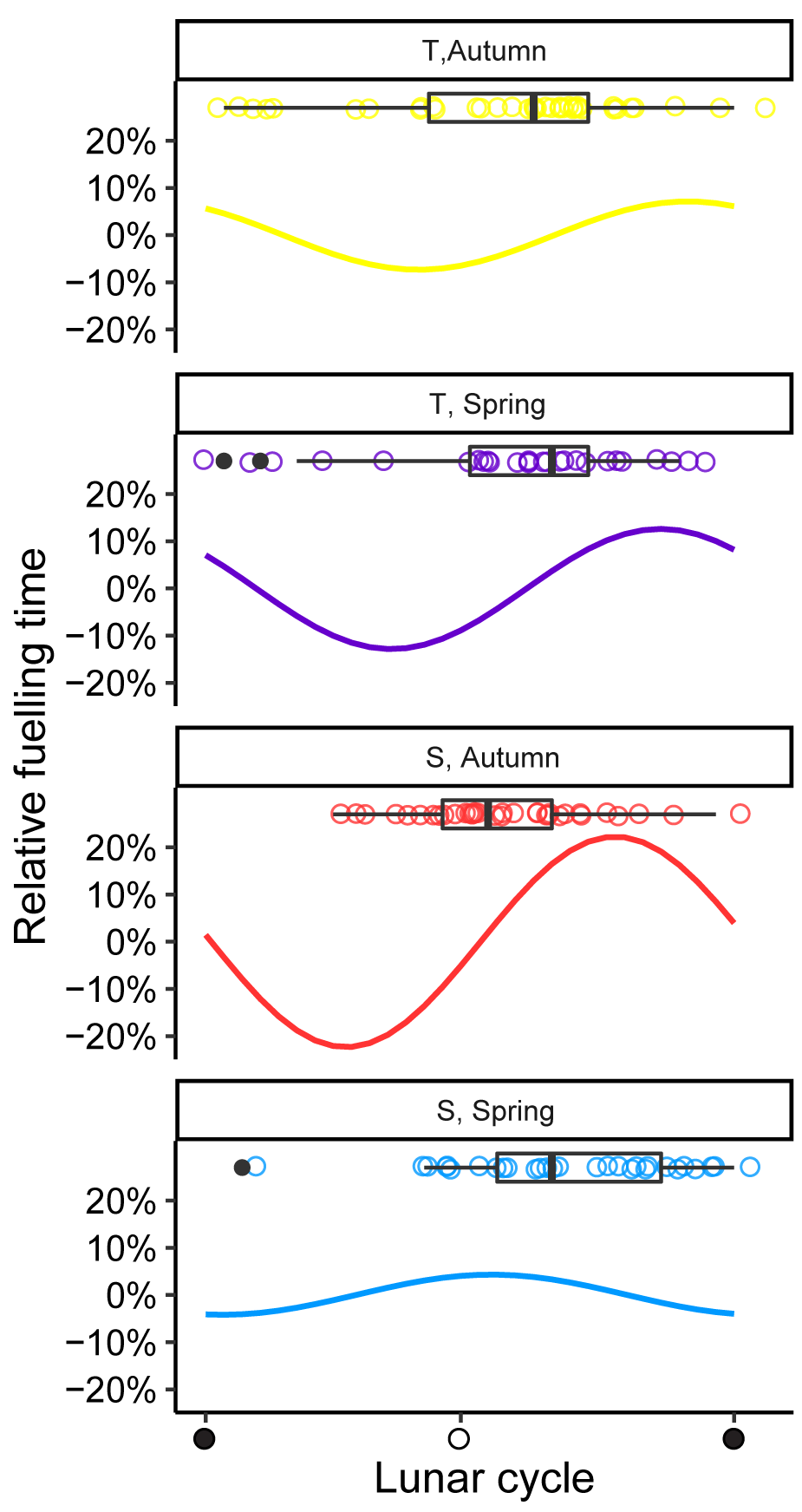

Supplement: S2 Fig — Bars indicate median departures, boxes indicate 25th to 75th percentiles, whiskers indicate ranges, and black dots indicate outliers. Lines represent the relative difference in cumulative foraging time under median stopover durations for each region (Temperate and Sahel zones) and season (autumn and spring), respectively. Filled and open circles on the x-axis represent the timing of new and full moon, respectively. The effect of optimal timing relative to the lunar cycle is expected to vary depending on the duration of the stopover. With the estimated increase in daily foraging duration during moonlit nights, an optimal timing of an autumn stop in the Sahel zone with a median duration of 17 days would increase the cumulative foraging time 22% relative to average. In contrast, for a spring stop with a median duration of 33 days, the corresponding gain is only about 4%. For the median stopover durations in the Temperate zone of 22 and 25 days for spring and autumn, respectively, optimal timing would result in intermediate gains. As stopover duration increases, the optimal departure timing relative to the lunar cycle will shift. Contrary to expectations, median departure timing was rather similar between sites occurring 2–8 days after full moon. Underlying data are found in S1 Data. (TIF) [file pbio.3000456.s007.tif]

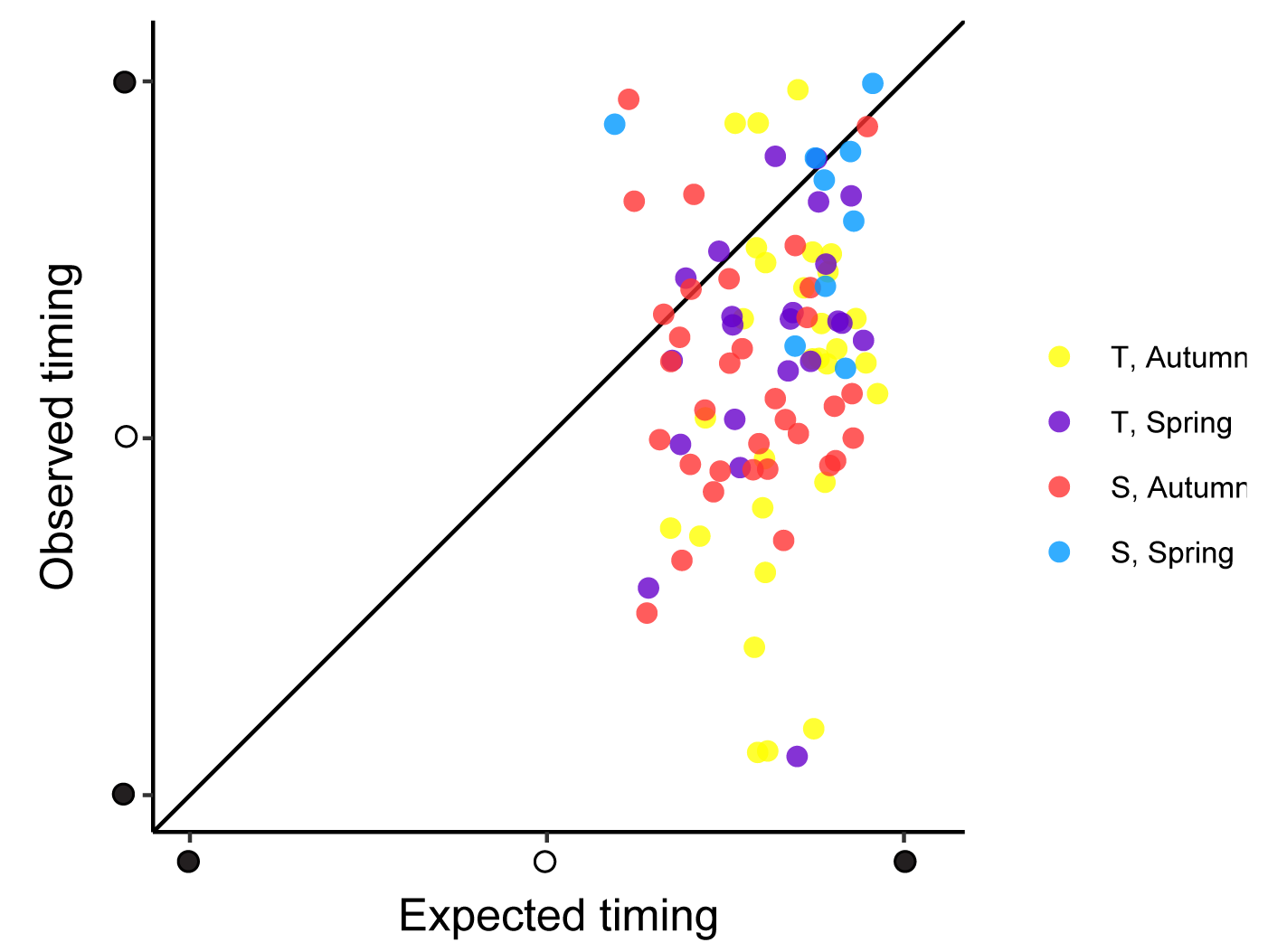

Supplement: S3 Fig — Observed versus expected timing of departures in relation to lunar cycle (filled circles = new moon; open circles = full moon). Solid line represents “Observed” = “Expected” and colors represent stopover zone (T = Temperate; S = Sahel) and season, as in the main text. Overall, birds departed earlier than expected if they would strive to maximize the ratio between foraging time and stopover duration. Filled and open circles on the axes represent the timing of new and full moon, respectively. Underlying data are found in S1 Data. (TIF) [file pbio.3000456.s008.tif]

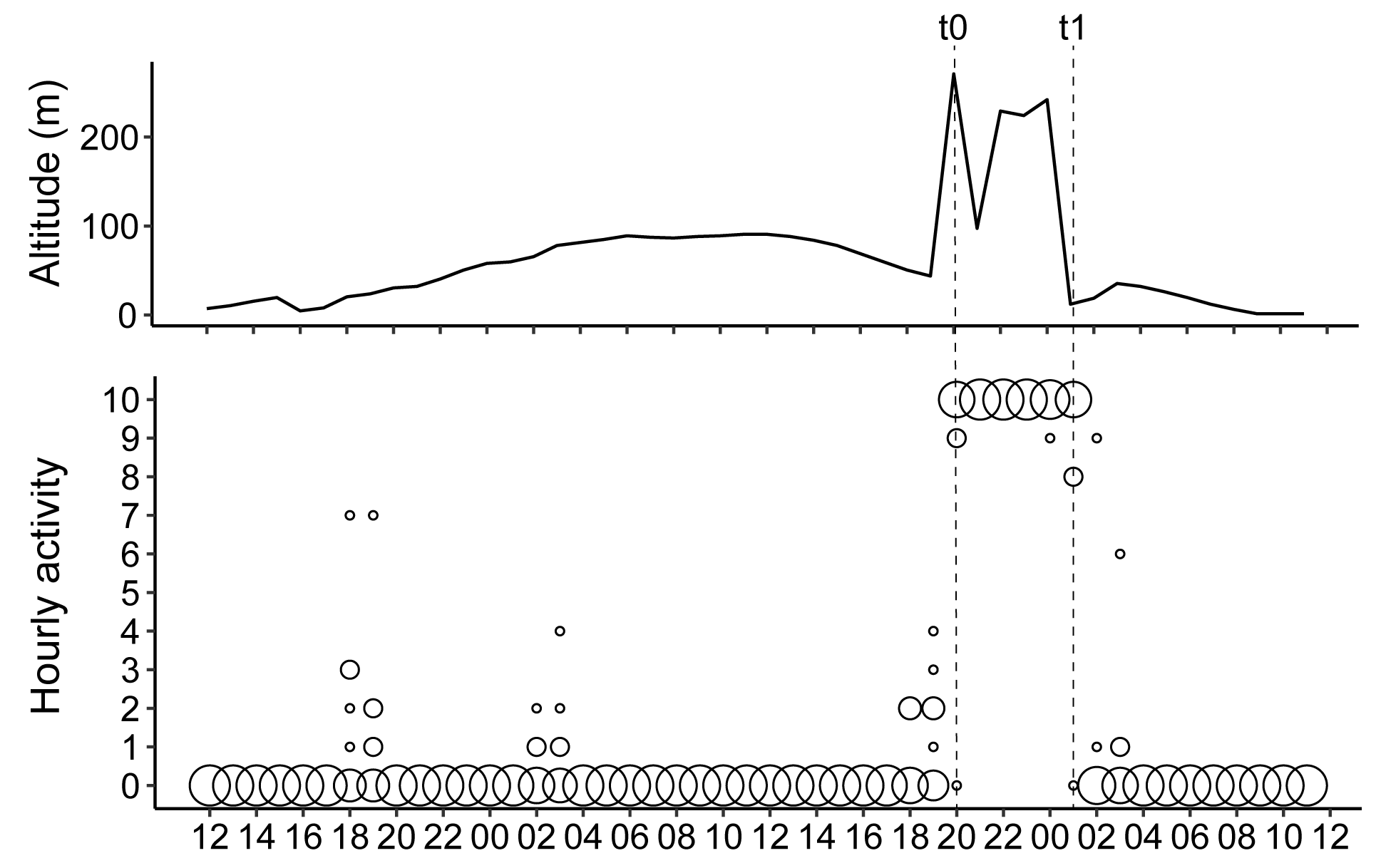

Supplement: S4 Fig — Hourly measurement of activity (bottom) and barometric-based altitudes (top) of European nightjar as sampled by a MDL-tag during a period of 2 days, starting and ending at noon. Circle size corresponds to the number of registrations observed per hour (1–12). An extended period of flight activity is initiated during hour 20 the second night (t0), resulting in a relatively large and rapid increase in altitude. At hour 01 (t1), the first low activity registration occurs, and the altitude has dropped and continues to be relatively stable throughout the rest of the night. This translates into a core period of 4 hours (21–00). As there are 11 registrations of high activity levels and only one registration of a low activity level both in the preceding hour (20) and the following hour (01), 55 minutes are added in both ends of the core period, resulting in an approximated flight segment of 5 hours and 50 minutes. Note that in both the first and the second night, there are several registrations of intermediate activity levels in the evening and morning, which likely are associated with foraging events. Also note how the altitude estimate gradually changes over the day as the sampled barometric pressure varies, likely due to, e.g., the passage of a low-pressure system. Underlying data are found in S2 Data. MDL, multisensor data logger. (TIF) [file pbio.3000456.s009.tif]

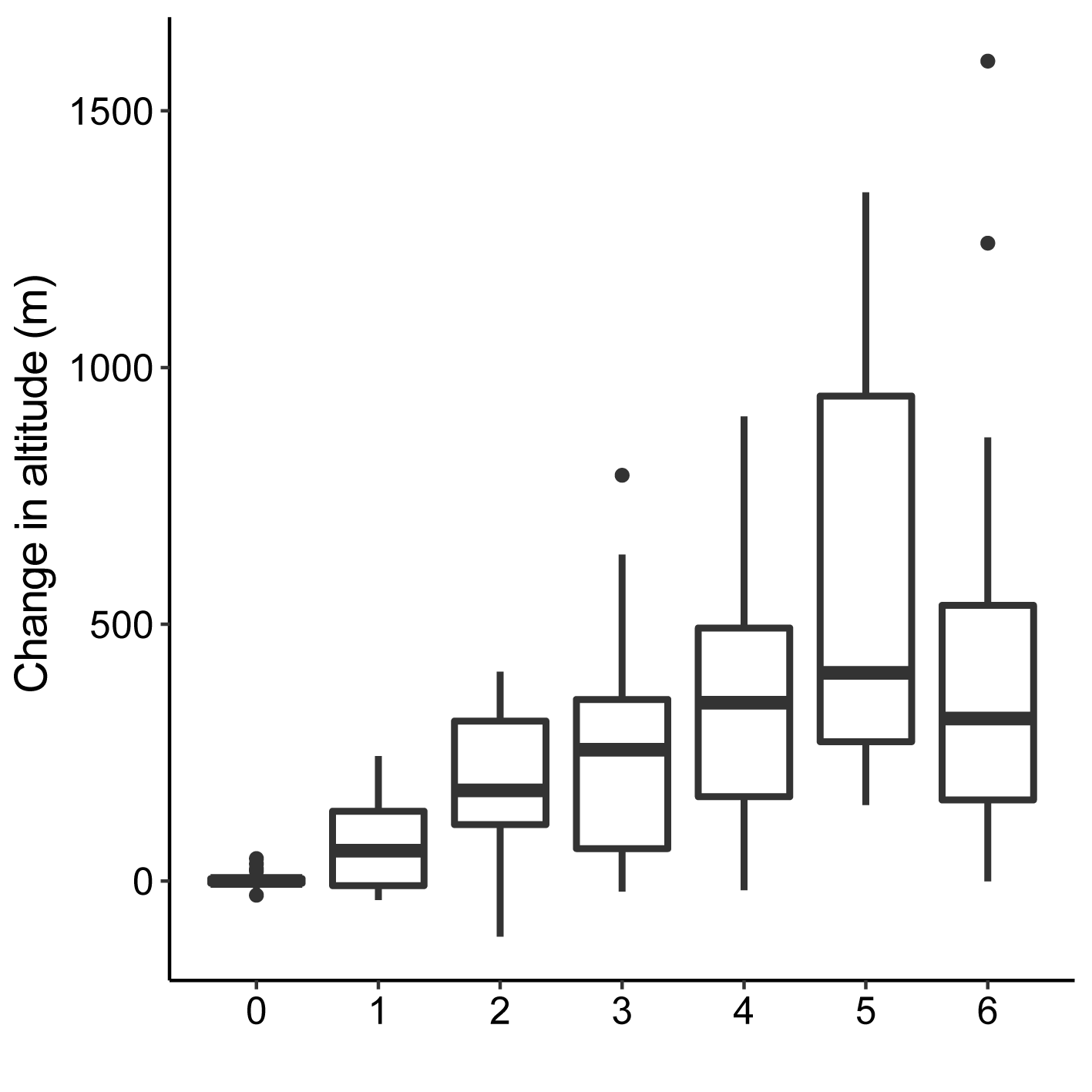

Supplement: S5 Fig — The barometric derived altitude measurement is taken hourly after the last of a series of 12 activity samplings. Each activity sampling consists of ten 5-minute registrations, and the number of positive activity registrations assigns it to one of eleven groups, 0–10; i.e., if there are no positive registrations in the activity sample, it is assigned to bin “0”; if there were three positive registrations, it is assigned to bin “3.” After one hour, all 12 activity samplings are distributed between the bins, and the activity pattern is stored. The change in altitude is the difference between the measurements taken during the pre-core hour and the hour before. Digits on the x-axis represent the number of 5-minute registrations during the pre-core hour, with values of 7 or higher indicating high activity. As the number of registrations increases, the change in altitude increases, indicating that high activity registrations at the initiation of a flight segment are part of the migratory flight. Underlying data are found in S2 Data. (TIF) [file pbio.3000456.s010.tif]
