# Supplementary material for: The lunar cycle drives migration of a nocturnal bird
Source: PLoS Biol. 2019 Oct 15;17(10):e3000456. doi: 10.1371/journal.pbio.3000456 (PMC6794068; doi:10.1371/journal.pbio.3000456)
Supplement: S1 Table — Start and stop date for each device used. Tag type refers to GLS, geolocation system GPS, global positioning system; MDL, multisensor data logger. (DOCX) [file pbio.3000456.s011.docx]

**S1 Table: Devices and sampling periods**

Start and stop date for each device used. Tag type refers to: global positioning system (GPS) and multisensor data-logger (MDL).

**Id Tag type Start Stop**

X13570 GPS 2015-07-28 2015-12-06

X13572 GPS 2016-05-30 2016-09-18

X13582 GPS 2015-07-01 2015-10-07

X13583 GPS 2015-07-06 2015-11-27

X13589 GPS 2015-07-23 2016-01-20

X15018 GPS 2016-08-01 2017-04-18

X15055 GPS 2016-08-07 2017-01-05

X15056 GPS 2016-08-07 2017-04-22

X20030 GPS 2017-07-21 2018-07-01

X20055 GPS 2017-08-02 2018-07-01

X20085 GPS 2017-07-20 2018-06-22

X20091 GPS 2017-07-14 2018-01-15

X20093 GPS 2017-07-16 2018-06-25

X20097 GPS 2017-07-16 2018-06-25

X20098 GPS 2017-07-16 2018-06-22

X20103 GPS 2017-07-20 2018-06-22

X20104 GPS 2017-07-20 2018-06-25

X500 MDL 2016-07-15 2017-04-27

X506 MDL 2016-07-15 2017-05-23

X523 MDL 2016-07-15 2017-05-19

X526 MDL 2016-07-15 2017-05-19

X531 MDL 2016-07-15 2016-12-24

X572 MDL 2016-07-15 2017-05-25

X627 MDL 2016-07-15 2017-06-18

X630 MDL 2016-07-15 2017-05-30

XD86 MDL 2017-08-01 2018-06-08

XD87 MDL 2017-08-10 2018-06-10
