# Supplementary material for: The lunar cycle drives migration of a nocturnal bird
Source: PLoS Biol. 2019 Oct 15;17(10):e3000456. doi: 10.1371/journal.pbio.3000456 (PMC6794068; doi:10.1371/journal.pbio.3000456)
Supplement: S2 Table — Linear mixed model with departure timing relative to lunar cycle as the dependent variable and the interaction between stopover duration and zone/season as the independent variable. Individual was set as random intercept (var = 14.26, SD = 3.788) and θ as random slope (var = 0.004, SD = 0.059). (DOCX) [file pbio.3000456.s012.docx]

**S2 Table**

Linear mixed model with departure timing relative to lunar cycle as dependent variable, the interaction between stopover duration and zone/season as independent variable. Individual was set as random intercept (*var* = 14.26, *SD* = 3.788) and *θ* as random slope (*var* = 0.004, *SD* = 0.059).

Effects Estimates SE df t value p

Intercept 18.41 1.854 55.93 9.927 <0.001

Dur:Temperate zone, aut -0.035 0.081 98.56 -0.431 0.667

Dur:Temperate zone, spr -0.031 0.093 110,47 -0.338 0.736

Dur:Sahel zone, aut -0.039 0.109 103.53 -0.362 0.718

Dur:Sahel zone, spr 0.054 0.059 78.78 0.926 0.357
