# Supplementary material for: The lunar cycle drives migration of a nocturnal bird
Source: PLoS Biol. 2019 Oct 15;17(10):e3000456. doi: 10.1371/journal.pbio.3000456 (PMC6794068; doi:10.1371/journal.pbio.3000456)
Supplement: Attachment — Submitted filename: Norevik et al. respons letter R3.docx [file pbio.3000456.s013.docx]

Dear Dr Norevik,

Thank you for resubmitting your manuscript "The lunar cycle drives migration of a nocturnal bird" to PLOS Biology. We noticed a few remaining editorial issues that will need to be addressed before your manuscript can be formally accepted. Your submission has been sent back to you to enable you to make these changes. You may have also received a set of production requirements as a separate email, please address the requirements in both emails before re-submitting your manuscript to us.

We would appreciate your prompt cooperation with our requests at your earliest convenience. If you have any questions or require clarification, please do not hesitate to get in touch.

*Authors’ response:* Thank you for the guidelines and comments that improve the quality of the manuscript. Below we provide detailed response to the requests of the Academic Editor.

The Academic Editor (AE) has assessed your revisions, and while s/he is largely satisfied, has asked us to transmit the following arising requests:

a) The AE would ideally like to see more evaluation of individual variation - you have added some statistical detail to the description of their modelling, but ideally the AE would like to see a more full-prose description of what the actual observed variation is.

*Authors’ response:* Although it is difficult to identify the source of the observed heterogeneity among individual birds, we agree that it is important to guide the reader towards potential causes of individual variation. We have added some explanatory notes regarding potential sources to individual variation to our results.

b) For figure 2b, the AE assumes that each dot corresponds to one individual? You should clarify this.

*Authors’ response:* We have changed the legend for figure 2b to clarify that each dot corresponds to one individual per day.

c) The handling editor noticed that the data displayed in Fig 4 had changed from the previous version; neither he nor the AE could find an explanation for the disparity between the last two versions of this Figure. The AE also said that in retrospect it is currently very hard to understand what exactly is shown in this Fig, nor how you can claim to measure "relative benefit" or infer optimality from a purely observational study. The AE feels that some more explanation, and/or a revised figure, is needed.

*Authors’ response:* We realised that when including the observations of and additional individual recovered in 2019 (as mentioned in previous cover letter) this accidentally introduced an error. We have now submitted a correct figure 4. Notice that it still differs slightly from the original figure due to the inclusion of the additional individual. We have also simplified the figure by focusing on the contrasting patterns expected if migrants depart as a response to diminishing daily foraging opportunities or if they strive as to maximise the available foraging opportunities by centralising stopovers around the full moon period. We now hope the figure is easier to understand.
